# A Design of the Resilient Enterprise: A Reference Architecture for Emergent Behaviors Control

**DOI:** 10.3390/s20226672

**Published:** 2020-11-21

**Authors:** Rob Bemthuis, Maria-Eugenia Iacob, Paul Havinga

**Affiliations:** 1 Department of Pervasive Systems, University of Twente, 7522 NB Enschede, The Netherlands; 2 Department of Industrial Engineering and Business Information Systems, University of Twente, 7522 NB Enschede, The Netherlands; m.e.iacob@utwente.nl

**Keywords:** cyber-physical systems of systems, systems of systems, cyber-physical system, reference architecture, emergent behavior, enterprise architecture, business logic, business rules

## Abstract

The sooner disruptive emergent behaviors are detected, the sooner preventive measures can be taken to ensure the resilience of business processes execution. Therefore, organizations need to prepare for emergent behaviors by embedding corrective control mechanisms, which help coordinate organization-wide behavior (and goals) with the behavior of local autonomous entities. Ongoing technological advances, brought by the Industry 4.0 and cyber-physical systems of systems paradigms, can support integration within complex enterprises, such as supply chains. In this paper, we propose a reference enterprise architecture for the detection and monitoring of emergent behaviors in enterprises. We focus on addressing the need for an adequate reaction to disruptions. Based on a systematic review of the literature on the topic of current architectural designs for understanding emergent behaviors, we distill architectural requirements. Our architecture is a hybrid as it combines distributed autonomous business logic (expressed in terms of simple business rules) and some central control mechanisms. We exemplify the instantiation and use of this architecture by means of a proof-of-concept implementation, using a multimodal logistics case study. The obtained results provide a basis for achieving supply chain resilience “by design”, i.e., through the design of coordination mechanisms that are well equipped to absorb and compensate for the effects of emergent disruptive behaviors.

## 1. Introduction

Though today’s enterprises are becoming increasingly resilient against unprecedented and unpredictable forms of disruption, the changing and uncertain nature of the real world continues to severely affect society. Earthquakes and floods [1], droughts and heatwaves, animal diseases, and periodic outbreaks of influenza viruses, punctuated by occasional worldwide pandemics [2,3,4,5], are examples of the damage done to nature, humans, and assets. Resilience plans are needed that strengthen communities to prepare and plan for, absorb, respond to, and recover from present and future sources of disruptions [6].

Enterprises, which are susceptible to disruptive events, need resilience. They are confronted with ever-present sources of risk, various dynamics, harsh environments, and malfunctioning system components. Typically, these circumstances are managed with enterprise-wide goals in-mind. In this paper, we use the terms organization and enterprise interchangeably and in the broadest sense. This means, it can take any form, ranging from a subdivision of a single organization up to any network of businesses (e.g., a supply chain) operating according to any coordination achieving a common business goal. As such, organizations have become complex socio-technical systems of heterogeneous systems (e.g., system of systems) that pursue their own—sometimes conflicting—goals, behaviors, functions, and requirements. The behavior executed by such local autonomous entities may, however, conflict with the system-wide goals. Despite these occasionally conflicting behaviors and goals, enterprises should have the ability to cope with emerging threats and to adapt to turbulent environments, while satisfying stakeholders’ needs. For example, organizations are encouraged to implement mechanisms to support their absorptive capacity [7]. Understanding how to mitigate such risks is imperative to achieving long-term business objectives.

In this context, given the unpredictability of both the environment in which an enterprise operates and the internal behavior of the autonomous components that make up an enterprise, the management of resilience may involve shifting from the classic a priori (holistic and) efficient planning towards a more real-time response with continuous (micro)adjustments. We are shifting towards a System of Systems (SoS) mindset, in which the consideration is no longer merely the computer or human, but interconnected Constituent Systems (CSs) [8]. An SoS is an “integration of a finite number of constituent systems which are independent and operable and which are networked together for a time to achieve a certain higher goal” [9]. A CS is “an autonomous subsystem of an SoS, consisting of computer systems and possibly of controlled objects and/or human role players that interact to provide a given service” [10]. Such CSs operate autonomously and may need to change their plans continuously based on the SoS’s situation. Instead of managing a whole SoS holistically and in a fully predictable manner, CSs have their own goals and planning that enables them to act on emergent behaviors as soon as they arise. To be able to make an SoS resilient against disruptions, micro-behaviors as modeled in CSs, should be balanced against the higher-level (macro)behaviors of the SoS.

An awareness of the physical environment is crucial in that regard. The more we are aware of, for example, physical and chemical processes, the easier it may become to grasp emergence propagated deeper into an SoS. To this end, modern sensors can convert properties exhibited in nature into electrical output signals in a fine-grained manner. This gathered data can relevant for any CS in an SoS. Therefore, it is not enough anymore to study sources of emergent behaviors in isolation. Disruptive emergent behaviors may be identified and monitored on a near-physical level at one CS, which can be important for another CS. Thereby, we arrive at the notion of a Cyber-Physical System (CPS). A CPS comprises interconnected, yet autonomous, physical assets and computational capabilities [11]. This means the joint dynamics of SoS and CPS should be studied together, and this is what sets the emerging discipline of Cyber-Physical Systems of Systems (CPSoSs) apart from these individually established fields. In this study, we focus on CPSoSs (Section 3 elaborates more on CPSoSs) for the detecting and monitoring of emergence in enterprises.

Ideally, features of a CPSoS controlling the real-time execution of operational (business) processes should be incorporated into the design of enterprises. To this end, the enterprise architecture (EA) discipline can help organizations to steer toward the desired state that embeds the CPS and SoS requirements. EA deals with the description of an enterprise from an integrated business and IT perspective that is intended to narrow the gap between business and IT stakeholders and improve business and IT alignment [12]. However, classic EA models fall short in this respect, as EA models give a rather high-level static abstraction of business and IT components. More recently, attention has been paid to physical components. EA modeling languages such as ArchiMate [13], recently adopted concepts to also describe entities in the physical domain [14]. The extension with concepts for Operation Technology (OT) modeling allows the describing of emergent behaviors that are close to established (physical) workflows. That is, it becomes possible to use EA models to adapt organizations to measure and optimize the recovery before or during the occurrence of a disruption at the physical layer. Later, suitable mitigation strategies can be incorporated into the different layers of an enterprise, enabling the movement beyond the survival of disruptions only and the achievement of true business-IT-OT alignment. For example, by embracing adaptive EAs and advanced decision support systems, an enterprise can gain from emergent behaviors instead of suffering losses. We advise that, the sooner emergent behaviors are detected, the sooner preventive measures can be taken to ensure the resilience of business processes execution. Thereby, a CPSoS approach plays a key role in establishing enterprises that are resilient against disruptive emergent behaviors.

Designing architecture and model-based analytics that ensure a fair symbiotic interaction between micro- and macro-behaviors and objectives can be challenging. This is a key concern addressed in this paper by keeping resilience in mind. To this end, upcoming technological advances propagated by Industry 4.0 and CPSoS initiatives should make emergent behaviors more explicit through the generation of real-time operational data that can be used to monitor and manage such complex systems (such as supply chains and business networks). Model-based and data-driven approaches, such as the notion of a digital supply chain twin for managing disruption risks and resilience [15], can support the integration of physical and cyber worlds. However, although capability enhancements with Industry 4.0 initiatives may be in the offing, manifested through, e.g., lower costs, improved quality, or increases in speed [16], it is evident significant challenges are yet to be overcome. Efforts are needed to address Industry 4.0 challenges on scientific, technological, and societal issues, including aspects of technology, security and privacy, and standardization [17]. Interoperability, integrability, and modularity issues [14] are some of the key concerns we are addressing in this paper.

The main goal and novel contribution of this paper is to design a reference EA embodying the integration of distributed CSs (containing its own business logic) and central control mechanisms, and thus realizing a symbiotic relationship between autonomy and central control that coexists within an enterprise. We argue that such an architecture inspired by biological systems (e.g., beehives and ant colonies) is more likely to be successful in achieving resilience than both the classic hierarchies and architectures with distributed control. To achieve this, we put some inspiration from Industry 4.0 initiatives, which are expected to bring advanced integration systems (e.g., between a physical and digital system) [18].

The EA proposed in this paper gives an overview of the functional building blocks that are required to support the execution and control of business logic for detecting and monitoring emergent behaviors. Central in this research is the embodiment within a CPSoS context as emergence is at the core of CPSoS thinking [10]. As there is a vast and scattered amount of literature on emergent behavior components for architectures, we conduct a Systematic Literature Review (SLR) to discuss relevant and currently used architectural building blocks and to derive architectural practices. We supplement these findings with our (work-in-progress) efforts on such designs [19,20]. More precisely, we show how enterprise-wide behavior and goals can be united with the behavior of local autonomous entities, aiming for an adequate reaction to disturbances. By doing so, we illustrate how disruptive emergent behaviors can be compensated in order to pave the way for a resilient enterprise. We demonstrate the proposed architectural design (the artifact) and some of the capabilities via a proof-of-concept implementation using a multimodal logistics case study.

In summary, we believe that the following contributions lay a good foundation for achieving resilience “by design”, which can address the important issues identified earlier; (i) a literature study on EA requirements for monitoring and detecting emergence, (ii) a reference architecture that extends the known models with architectural artifacts and their relationships, and (iii) a demonstrated proof-of-concept implementation showing the effects on the resilience of an enterprise in a logistics case study.

The remainder of this paper is structured as follows. Section 2 details the research design followed in this paper. Section 3 provides background, shedding some more light on emergent behaviors with respect to Industry 4.0 and CPSoS, and the context of resilience. In Section 4, we report our findings on requirements for the reference architecture. Section 5 presents the reference EA. Section 6 addresses the prototypical implementation, illustrated with a case study. Section 7 provides a discussion. Finally, Section 8 concludes and gives an outlook on future work.

## 2. Research Design

In this paper, we follow the design science research methodology (DSRM), as proposed by Peffers et al. [21], because it is widely used in information systems research and provides clear guidelines and an iterative approach for designing and testing an artifact. Our research objective (see Section 1) indicates the development of a reference architecture as an artifact, of which we distill requirements by identifying the current state of the art. For this purpose, we apply a multi-method approach consisting of a SLR nested into a design science research cycle.

**Figure 1:**
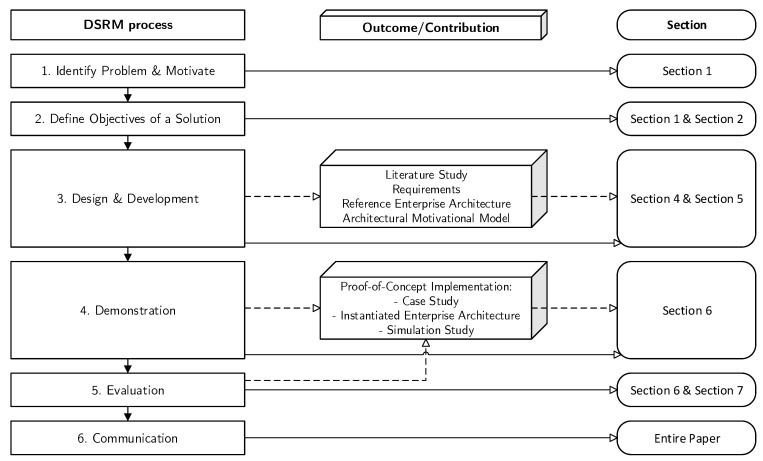
Research methodology.

The research methodology followed in this study, shown in Figure 1, is as follows. The problem identification and motivation, as well as the research objective, are addressed in Section 1. The proposed artifact in this study is a reference architecture is mainly based on architectural components reported in current scientific contributions. In this study, the term architecture is used in a broad sense and refers to “the fundamental organization of a system embodied in its components, their relationships to each other, and to the environment, and the principles guiding its design and evolution” [22]. To this end, we reuse existing architectural components by conducting a SLR. Furthermore, we build further on our own work-in-progress efforts [19,20]. To guide the collection and review process, we conduct a SLR by following the guidelines as proposed by Kitchenham [23], Kitchenham et al. [24], and Rouhani et al. [25]. Thus, one intermediary artifact prior to the design and development of the reference architecture is the reporting of relevant literature. The SLR protocol is further documented in Section 4.

The requirements derived from the SLR and our work-in-progress efforts are used to design and develop the architectural reference model (our main artifact), reflecting the state-of-the-art. An instantiation and use of this architecture are applied to a logistics case study in the form of a proof-of-concept implementation. This demonstration shows some of the architectural capabilities and provides some evidence of how an enterprise may achieve resilience.

Although the contributions of this paper are domain-independent, we devote special attention to the transport and logistics domain throughout this research. In general, this domain encompasses the transport of goods, commodities, and services in the markets for goods, services, and raw materials. One reason for this interest is that the united interaction, as explained in Section 1, becomes tangible when talking about material goods. For example, the local actor’s behaviors can be reflected in the business logic of logistics entities such as smart cargo or autonomous driving vehicles. Another reason for the special attention to this domain is that we contribute to the research line focusing on supply chain logistics, as stipulated in a doctoral research plan of Bemthuis [26]. We argue that the smart supply chain’s IT infrastructure is a key enabler for managing interacting behaviors since it enables the independent control and coordination of various supply chain components. More broadly, our work stresses on avenues for addressing the aforementioned challenges that are present or could emerge in the near future within a CPSoS context, such as multimodal transportation, large communication networks, and electric grid infrastructures.

## 3. Background

This section gives some background information on topics of this study. Section 3.1 addresses background knowledge on emergent behaviors. Section 3.2 proceeds with addressing the notion of resilience. Finally, Section 3.3 discusses the combined role of emergence and resilience.

### 3.1. Emergent Behaviors

Throughout the years, emergent behavior (also named emergence) has been examined in many contexts. Emergence can be described as the phenomena occurring on a macro-level, resulting from the action and interaction among micro-level components [27]. Many phenomena that arise out of complex, adaptive systems are called “emergent” [28]. Classic examples can be found in nature, such as bird flocks, fish school, and insect swarms, which frequently exhibit complex and coordinated collective behaviors [29]. Typically, the micro-level entities use only limited environmental information and simple rules (e.g., business rules), yielding unrivaled opportunities to link the action and behavior of individuals with dynamic group-level properties.

In particular, we believe that technological advances brought on by Industry 4.0 and CPSoS initiatives have become mature enough for capturing and anticipating on emergence. It would go beyond the scope of this paper to extensively elaborate on the concept of CPSoS. Here, for conceptual CPSoS theory, we refer to the book of Bondavalli et al. [10]. We use a definition of CPSoS as a SoS whose interacting CSs are CPSs, whereby a CPSoS manifests itself within a hybrid nature, consisting of physical components as well as controls, communication channels, and local- and system-wide optimization methods and management systems [30]. In an enterprise context, a CPS entails the integration of a real enterprise (in a physical space) and an artificial enterprise (in a cyber space) [31].

Typically, emergence is neither predictable, nor intended by design, which makes its managing challenging. Emergent behavior is also exhibited within a CPSoS. A CPSoS is characterized by intercommunications and interactions between many separate and autonomous components (i.e., CSs) that develop properties that are isolated from and cannot be traced back to the separate systems [32]. When focusing on the consequences and prediction of emergent behaviors of a CPSoS, we can distinguish expected versus unexpected emergence and beneficial, neutral, and harmful emergence, as shown in Table 1. Detecting and avoiding an unexpected detrimental emergent phenomenon is problematic [10]. However, as knowledge of CPSoS progresses, more and more emergent behaviors move from problematic to a positive surprise or prescribed design principle.

Following the classification proposed by Fromm [33], we can identify four types of emergence useful for CPSoS:

- **Type I: nominal or intentional emergence** is fully predictable due to the controlled and planned interaction of the individual components. Any level simpler than that of the whole CPSoS cannot comprise the analysis of this type, which makes it challenging to detect or predict emerging behaviors from inside the system. To exemplify, consider a freight barge of which a cargo attribute (e.g., a smart pallet) is not able to comprehend the barge’s emergence.
- **Type II: weak emergence** comprises systems with top-down feedback, imposing constraints on the local interactions. This type of emergence is in principle identifiable, but the interaction complexity can be prohibitive for extant analysis techniques. Consider interacting systems with a (co-)dependency (e.g., a scarce resource) resulting into cumulative, sometimes predictable, propagation across the whole CPSoS. The formation of traffic jams is an example.
- **Type III: multiple emergence** is characterized by multiple positive (expansion impossible) and negative feedback (contraction impossible) loops in complex systems. New systems can appear while old ones disappear. The behavior (of CSs or of their environment) is non-deterministic and can be chaotic. An example includes truck platooning or the bullwhip effect.
- **Type IV: strong emergence** comprises completely new properties that cannot be predicted, even in principle, to cumulative system effects. The macro-level properties are irreducible to elementary parts and their interactions [34]. Any attempt at explaining this type of emergence would be rendered futile because of combinatorial explosion. Classic strong emergent phenomena are life and culture.

Existing approaches offer little basis for investigating the development and behavior of a macro-entity that is formed and keeps developing in complex systems under the presence of manifold CSs. As stated by Van Lier [32], eventually a CPSoS enables the creation of its own feedback loop between the global level of the CPSoS and the individual level of the separate autonomous CSs. In turn, understanding self-organization, entanglement, and emergence could make a key contribution in forging innovations [32]. In this line of thinking, we foresee that a complex sociotechnical SoS, such as a CPSoS, empowers our envisioned united relationship between the cyber space and physical world, and, consequently, give rise to modeling new approaches for understanding emergent behavior. We do not expect that CPSoS alone will provide an all-encompassing type of solution to all challenges regarding emergence, especially not regarding the tackling of strong emergence (and of many simpler forms of emergence). However, it may enable new forms of macro- and micro-level features inherited from emergent processes. New rules and patterns can appear that could be captured and understood by the physical context and the interactions between its environment, which may also cause ubiquitous scale effects on enterprises. Taken together, models and modeling approaches are needed that facilitate understanding of emergent behavior aspects as an integral part of an architectural enterprise design.

Detecting, monitoring, and controlling emergent behaviors in a CPSoS may be possible but in limited terms. A first step in dealing with emergence is to detect that they exist [35]. This may be done through data analytics methods, such as simulations. A second step is to keep track of the detected emergence via a monitoring mechanism. Expected and unexpected deviations in a CPSoS may then be spotted. Notice that the detecting and monitoring capabilities themselves may also be part of the emergent behaviors of a system. The third and final step is the emergence control (or managing) part, which comprises defining actions that changes parameters to sustain a particular emergent behavior [36]. Various control approaches are mentioned by Parunak et al. [35] and Mittal and Rainey [36]. We put forward that an effective (CPSoS) emergent behaviors control can be achieved once the detecting and monitoring of emergence as well as the planning and execution of (corrective) actions are part of the system design.

### 3.2. Resilience

The concept of resilience has gained popularity among widespread domains, as demonstrated by the already large body of literature available. This popularity has also revealed some confusion about the use of the term resilience and the associated abundance of metrics and indices [37]. Here, we discuss some core elements of resilience thinking and take some steps to discuss resilience within the context of CPSoS and enterprises.

A classic definition of resilience is given in the field of ecology by Holling [38] and constitutes a measure of persistence of systems and their ability to absorb change and disturbance and still maintain the same relationships between system components. This perspective, which is often named ecological resilience, emphasizes the system’s ability to persist in the original state subject to disturbances. The term resilience has also been used in a narrower sense, referring to the ability to return to an equilibrium state after a disturbance. This viewpoint can also be named as stability [38] or engineering resilience [39]. Notice that engineering resilience in complex systems suggests that a disruption can bring a system over a threshold that marks the limit of the stability domain of the original state [40]. As such, complex systems, such as CPSoS that exhibit multiple CSs, can cause the system to be attracted to a different state. In this narrower definition, the focus is more on the system’s ability to return to a convenient state following disruptions, while it would also be interesting to resist change in the face of disturbance. In a broader term, Masten [41] describes resilience as the capacity of a system to adapt successfully to perturbations that threaten system function, viability, or development. This definition stresses on threats that can undermine a system, implying the involvement of risk management. In the context of disaster management, resilience can include readiness, response, and recovery strategies [42]. According to Fiksel [43], resilience is the capacity to survive, adapt, and grow in the face of turbulent change. This definition places emphasis on an evolving nature, which can also be of importance within a CPSoS. For more explanatory theory on resilience, we refer the reader to the works in [44,45,46,47,48] and for more work focusing on resilience within an enterprise context we refer to the works in [49,50,51,52].

Thus, the term resilience has been broadened in recent years and comprises a multitude of concepts. We agree with Hodgson et al. [37] that capturing resilience in a single metric might not be feasible but that plural features that make some systems more resilient than others can be measured. For example, Petit et al. [53] proposed a resilience index based on the criteria robustness, resourcefulness, and recovery, with sub-criteria. Furthermore, the dynamics of an SoS makes it challenging to anticipate its behavior at design time. Therefore, (CP)SoS quality requirements can be difficult to address [54,55]. We take up the conclusions to be drawn for the notion of resilience with regard to emergence and CPSoS in the next subsection.

### 3.3. From Emergence to Resilience

While the aforementioned studies give a good indication of the importance of considering disruptions, risks, and adaptability when discussing (or defining) resilience, they do not provide guidance on how to conceptualize this within the realm of emergence, CPSoS, and enterprises. Below, we shed some light on the notion of resilience in that regard.

A consequence of the increasing interrelationships, interdependencies, and thus tight coupling between CSs and processes is the realization of highly efficient organizations. At the same time, this leads to an increased susceptibility to disruptions and to ripple effects toward other systems or organizations. Key in enabling resilient enterprises is to design antecedent conditions which contain both inherent resilience as well as inherent vulnerabilities by understanding properties and functions that target the system itself and the source of disruption. This vision can be termed as emergent resilient by design. The design part refers to SoS that are able to target the system itself and also the source of disruption.

In order to offer a common definition on resilience, we draw upon the insights from the previous discussions and adopt a definition from social-ecological systems theory [56,57] and a United Nations report on risk management [58]. These fields of study predominantly involve intersecting systems which shape and construct communities, which seems a natural candidate for transition to CPSoSs and enterprises. To this end, we define resilience as the ability of a complex system, such as an enterprise CPSoS, to anticipate and adapt to change using its own inherent capabilities to absorb impacts and to participate in the enterprise processes that support the system in reorganizing, changing, and learning from the event, all in a timely and efficient manner.

Resilience within an enterprise CPSoS is the kind of resilience achieved by an imaginary frictionless pendulum (i.e., integration) weaving the behavior of local autonomous entities with the enterprise-wide behavior, targeting sources of disturbances while evolving the CPSoS in a dynamic way. Enterprise resilience is thus an emergent property of a complex system. Similar to the previously made classification of emergence types, one could decompose emergence within an enterprise resilience context (e.g., weak emergent enterprise resilience) by referring to resilience features of an enterprise. We base a measurement means on a report from United Nations International Strategy for Disaster Reduction (UNISDR) [58] and determine the resilience of an enterprise with regard to potential disruptive events by the degree to which the enterprise has the required resources and is capable of self-organizing both in anticipation of and during times of need.

## 4. Requirements for the Enterprise Architecture

This section describes the requirements for the EA. First, we discuss how the SLR is conducted. Then, we discuss the findings, after which we report on the distilled requirements.

### 4.1. Systematic Literature Review

To identify relevant studies that address existing architectural components and to identify effective architectural practices for modeling emergent behaviors, we have performed a SLR. A SLR is a methodical way to identify, evaluate, and interpret the available studies conducted on a topic, research question, or a phenomenon of interest [23]. This method is chosen because it reduces researchers’ bias when performing this review by adhering a review protocol. The methodologies described in [23,24,25] inspired us for conducting our review, because they have a clear focus, provide practical guidelines, and are often used by other researchers in the field. We report here on the methodology part and communication part of the SLR.

#### 4.1.1. Research Questions

The research questions that we intend to answer in this SLR investigate the functionality of architectural components addressing emergence in selected primary studies. By doing so, we aim to derive effective factors (e.g., items or quality attributes) that are used for modeling emergent behaviors in a CPSoS context. The research questions (RQs) are as follows.

RQ1. Which main emergent behavior functionality have been addressed in the selected publications?
RQ2. How were the relevant architectural components for addressing emergent behavior in the selected studies evaluated?

#### 4.1.2. Search Strategy

The main strategy employed in our SLR study was to first find a preliminary set of primary studies by a broad search and, subsequently, to narrow down the results by applying predefined (quality) criteria. Our strategy concentrates on searching in scientific databases rather than in specific books, technical reports, or case studies repositories, as we assume that the major research results in these sources are also usually described or referenced in scientific papers. Searching broadly in database sources, as recommended by Kitchenham [23] and Kitchenham et al. [24], and utilizing electronic databases, the databases as listed below have been selected to perform the search process. Due to the high number of duplicates, we decided to not add more search sources to the list because we expect that the number of relevant papers will not significantly increase.

As search keywords, we composed a query based on three main parts, mainly derived from Section 1 and Section 3, that are complemented with synonyms and linked with AND operators: (1) emergent behavior, (2) architecture, and (3) CPSoS. In addition, we complemented the third keyword with the terms internet-of-things and industry 4.0. We used the following query; (“emergent behavio*” OR “emergence”) AND (“architecture” OR “reference model” OR “framework”) AND (“cyber-physical system*” OR “cyber physical system*” OR “industry 4.0” OR “internet of things” OR “internet-of-things” OR “system of system*” OR “system-of-system*” OR “system*-of-system*”).

A justification for the inclusion of these three keyword compositions is as follows. The first part of the keyword composition covers emergent behaviors, because one of the key focus areas of our study is about detecting and monitoring emergence. We could have also included related terms, such as agents as those constructs are often linked to the manifestation of emergence [59]. However, given the popularity of agent(-based) and some other approaches, there is a vast and scattered amount of literature available, which may not focus that much on emergence. For example, regarding the use of the term “agent” there is no universal agreement on the definition [60], leading to a widespread amount of literature. At the same time, we expect a burden amount of literature if we search on broader concepts of emergence, such as properties mentioned by Goldstein [27]. Instead, we decided to explicitly include “emergent behavior” and “emergence” to cover an umbrella of technological concepts (e.g., agents, holons, etc.) that can be associated with emergent phenomena.

The second part of the search string focuses on extracting building blocks for the future architecture, from literature. Given the enormous breadth of literature on elements that may be of importance when modeling emergent behaviors, we limit the research to more mature literature that goes beyond the conceptualization of a single component and takes a next step towards a whole architecture. Thus, the terms “architecture”, “reference model”, and “framework” are added to constrain the search results and ensure the inclusion of sources that focus on an interlinked set of defined concepts. This helped us restrict the focus of interest to a manageable and sound search for literature.

The third part of the search string composition focuses on limiting the search to literature about SoS, CPS, and CPSoS. These keywords are chosen because we want (1) to reuse existing theories on integrated distributed CSs (for motivation see, e.g., Section 1); (2) to incorporate relevant requirements sources; and (3) to avoid unnecessary efforts for identifying, understanding, and correlating applicable CS requirement sources on a project-wise basis. In addition, we extended this part of the search string with the terms “Industry 4.0” and “internet of things”. There is chosen to include these additional terms to expand the potential knowledge base with some recent technological advances. These terms are inherently associated with: (1) (large-scale) interconnected systems, (2) business logic delegated to distributed parts of the system, and (3) entanglement of the physical and cyber world. In particular, fundamental concepts of Industry 4.0 relate to CPSs and decentralized self-organization [18]. Besides that, the term Industry 4.0 is commonly associated with the trend to acquire more data in a CPS context [61]. Last, SoS, CPS, and Internet of Things are often interchangeably used in literature [62,63].

Please note that the term “resilience” is not included in the search string. As discussed in Section 3.2 and Section 3.3, we specified resilience as an emergent property of a complex system and that it is difficult to capture resilience within a single term. Therefore, we left out this further demarcation in the literature search for future studies. Notice, however, that there is some evidence that the contribution of Industry 4.0 initiatives are positively impacting the resilience of supply chains [16]. This may also be translated to wider perspectives, such as enterprises.

By this search string, we are aware that we might already exclude relevant papers in the beginning by not including more synonyms, such as “system structure” as substitute for “architecture”. However, the present work is not meant to replicate exhaustive SLRs but to build on commonly used design principles or best practices to arrive at a set of unifying principles that account for the phenomenon of emergent behavior across disciplines and systems. Furthermore, as we calibrated our search string in a pilot search in a single database, we expect to mitigate the risk of not considering relevant contributions. Moreover, the SLR is not the main goal of this paper, but is just helping us in defining requirements for the reference architecture which we aim to design.

After applying the search string (on the 18th of May 2020) to find relevant studies in paper’s title, keywords, and abstract, we obtained the following initial results per search engine; IEEE Xplore: 254; Scopus: 669; Web of Science: 447. It is important to notice that some databases required minor changes in the search string. Efforts were made to ensure that the adjusted search string were logically and semantically equivalent. In order to be left with only the most meaningful literature, we applied the selection and filtering steps as described hereafter.

#### 4.1.3. Filtering the Initial Literature Search Results

The search strategy is proceeded by applying inclusion and exclusion criteria. The inclusion criteria were (i) studies that include on-line accessible full-text versions, (ii) fully written in English, and (iii) journal or conference papers. Consequently, duplicate articles were removed under the exclusion criteria: (i) studies with the same title and author found in more sources will be considered as one study, and (ii) if approximately the same topic is produced by the same author(s), only the most complete (or latest) version of the study is included. After conducting these steps, the first sample set contained 749 papers.

#### 4.1.4. Title, Abstract, and Keywords Screening

Subsequently, we assessed the papers on their relevance based on title, abstract, and keywords. Papers satisfying the following criteria were rejected; (i) not contributing to RQ1 and (ii) short contribution (e.g., smaller than or equal to 2 pages, a doctoral consortium contribution, or a contents guide or index, etc.). In the case of a doubt, we decided to not (yet) reject the paper. The results after this screening is 190 papers.

#### 4.1.5. Full-Text Screening

We proceeded with a full-text screening and eliminated papers that were not satisfying the exclusion criteria. For example, when the term emergent behavior is addressed in the further research part only. Again, in the case it was contentious whether to reject the paper, it was decided to not (yet) reject the article. This selection has yielded 77 papers. To further drill down to a core selection of papers, we applied a scoring assessment based on two criteria: (1) emergent behavior relevance and (2) SoS, CPS, or CPSoS relevance. Although a (CP)SoS intrinsically exhibit emergent behavior, we impose an explicit notion on this phenomenon because of the focus of our research. The papers were rated with scores ranging from 1 to 5 by screening the full content of the article: 1 point: no relationship; 2 points: discusses one aspect; 3 points: discusses several aspects; 4 points: proposes a partial architecture; 5 points: proposes a full architecture.

Assessment conflicts were solved by discussions among the authors. The results of both scoring criteria were merged and all papers with an aggregated score of at least 6 were included in the literature list. Complementing this list with our previous two articles [19,20] and four manually added papers [64,65,66,67], resulted into a total of 46 papers [19,20,64,65,66,67,68,69,70,71,72,73,74,75,76,77,78,79,80,81,82,83,84,85,86,87,88,89,90,91,92,93,94,95,96,97,98,99,100,101,102,103,104,105,106,107].

### 4.2. Data Extraction and Findings

We extracted several details about each paper including the maturity of the research, the quality attributes mentioned, and requirements. Here, we report on the findings regarding the first two aspects, thereby contributing to RQ2. The results of the data synthesis on the requirements (RQ1) are presented in the proceeding subsection.

We classified the selected articles from a maturity level perspective, as inspired by Guessi et al. [64]: (i) perspectives or position study, which positions new ideas or directions on architectural functional components (e.g., basic principles); (ii) ongoing study, which reports preliminary findings that require more detailed validation; and (iii) complete study, which presents validated research (e.g., case study or experimental research). We categorized the key contributions of the papers as (i) architecture (e.g., abstract design concept of services, components, interaction, etc.); (ii) framework (e.g., general or special purpose architecture designed to be extended); (iii) architecture description language; (iv) model; (v) ontology, taxonomy, or controlled vocabulary; (vi) secondary/tertiary study; and (vii) viewpoint (e.g., experience paper).

**Figure 2:**
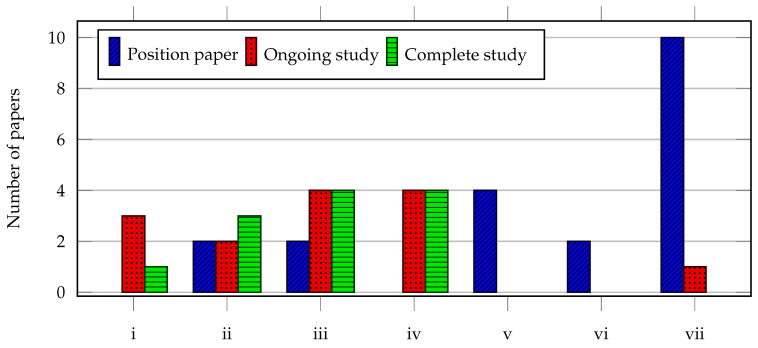
Research validation maturity. The contribution types are as follows; i = architecture; ii = framework; iii = architecture description language; iv = model; v = ontology; taxonomy, or controlled vocabulary; vi = secondary/tertiary study; vii = viewpoint.

Figure 2 shows the categorized papers according to their main contribution. Of the 44 primary papers, only 12 (27%) papers were considered as complete studies, which indicates a need for more research. Nevertheless, a total of 26 (59%) papers concerned either an ongoing study or a complete study, which implies that there is some mature basis from whereon we can build further. Furthermore, it is interesting that several papers [19,20,77,78,79,86,93,95,96] use agent-based modeling techniques to support their work.

Two studies summarized or synthesized literature in, respectively, a secondary [64] and tertiary literature study [84]. We report on some of their findings to shed some light on deeper and ongoing discussions. The work of Guessi et al. [64] discusses a SLR on SoS architecture description languages. Similar to our findings, their study indicates a lack of mature research with respect to validation of proposals and achieving a better understanding on the requirements of SoS architectures. Furthermore, we agree with their proposition that new CSs and purposes can emerge dynamically in a way that it was not predicted at design time, yet that many challenges faced for developing SoS can be dealt with at the architectural level. Although their study focused on SoS (software) architecture descriptions, we can put some inspiration from their findings to complement our study. Cadavid et al. [84] provided a tertiary study on SoS architecting by evaluating 19 secondary studies. They present a concept map to reflect a community-wide understanding of the concept of SoS and its relation with other commonly used terms. In our reference architecture construction phase (Section 5), we draw some inspiration from their suggested overview.

### 4.3. Requirements

Architecting SoSs and CPSoSs involves making numerous architectural design decisions. Especially for the implications of these decisions on effectively managing emergent behaviors. To delineate the relevance of our contribution, we focus on requirements that postulate the use of integration-oriented architectural styles (i.e., the united relationship as discussed in Section 1) for the execution and control of business logic. These requirements were established by both developing an understanding of CPSoSs (e.g., as discussed in Section 3) and reviewing existing literature. Based on discussions among the authors and project partners of this paper, we elicited and synthesized the requirements.

We first present some requirements that are considered as inherent to SoSs and mentioned by several of the analyzed papers. These requirements, as reported by the often cited work of Maier [108], Sage and Cuppan [109], and Boardman and Sauser [65], are listed below.

**Operational independence:** CSs can operate independently, fulfilling business logic purposes on its own. Essentially, this business logic contains an assignment of tasks in an enterprise to keep it running smoothly. Each CS has a set of duties that must be performed under the CS’s umbrella, where it is allowed to operate. The often dynamic business rules are the inherent commitments of the CS, and they are rules of what kind of enterprise it is operating and what kind of actions would be taken given this information.

**Managerial independence:** CSs not only can (i.e., operational independence requirement), but also do, operate independently. The managerial control can be directed (built and managed to fulfill a particular purpose), collaborative (e.g., CSs can partially collaborate voluntarily), and virtual (no central management over the SoS) [108]. Yet, CSs are autonomous with respect to their operational and managerial independence. A SoS can have several (business) rules governing the actions and behavior [88]. CSs can decide independently on whether they want to be part of an SoS or not. For example, federations among CSs may be shaped, such as temporary alliances of self-driving cars in truck platooning, contributing to superior organization’s goals.

**Distribution:** CSs can be physically dispersed, but they may communicate with other CSs. Mediators must enable, among others, communication and coordination to support inter-operations [73,83,103]. A mediator orchestrates resources (e.g., data, people, machines, computational power, etc.) across a variety of CSs, attempting to keep up with the demands placed on it. A mediator can achieve a specific emergent behavior by intervening the interaction [72].

**Evolutionary development:** A SoS is continuously changing in which CSs may appear or disappear. For example, the structure, function, and purpose of a CPSoS modify (e.g., extend, shrink, evolve, etc.) as time progresses. In turn, a CS is capable to dynamically adapt or evolve. This must be enabled at design- and run-time subject to the evolutionary development of the SoS. Furthermore, CSs can be at different evolutionary states in a CPSoS. This requires a form of multi-version evolution [10]. For instance, CSs should be able to interact with older versions of themselves. This also involves a CS life cycle management capability.

**Heterogeneity:** CSs are widely diverse in its capability, spanning varying operating systems, protocols, and communication methods. For example, such systems may have various elementary dynamics that operate on different time scales [78].

**Emergent behavior:** CSs united collaborating together results in unique functionality and emergent behavior exhibited at various CPSoS levels. Details on how the properties of emergence are applicable to a SoS should be included [84]. Emergent behaviors may appear in a CPSoS’s environment or because of an evolutionary step that changes how CSs interact. The CPSoS should be able to detect, monitor, and adapt based on emergence. More precisely, features may include components from a Monitor, Analyze, Plan, and Execute (MAPE) cycle. Besides that, a CS may attempt to stimulate emergence once the CPSoS prospers. This to learn from the event in order to prevent it from happening again or in a similar fashion at a later point in time and thereby (potentially) preventing an even bigger disaster. Likewise, a CS can sacrifice (parts of) itself for the well-being of a (CP)SoS. For example, emergence may be invoked by a prospering CS and, ultimately, cause an unexpected but beneficial outcome for an other CS. Attention should be given to mitigation strategies for minimizing occurrence and damage due to unexpected emergence. However, we should not create the illusion that all sources of emergence can be fully predicted and adequately dealt with. For example, (unexpected) detrimental emergence (see Section 3) can be a difficult one to reliably predict (if ever predictable at all).

Besides that SoS requirements are considered, we also pose several requirements of a CPS. Although the constructs SoS and CPS may seem to overlap as they are used in the same type of micro- and macro-scale systems [84], there are some distinct features that we impose in the following requirement.

**Cyber-physical system**: A computer system (the cyber system), a controlled object (a physical system), and possibly interacting humans are part of a CPS [10]. The encompassed computational and physical components are integrated and closely interacting to sense the changing state of the real world. A CPS should be able to directly record physical data using sensors and affect physical processes using actuators [67]. Furthermore, the CPS can evaluate and save recorded data, and actively or reactively interact both with the physical and digital world [67]. A SoS should also be operational without a CPS component. Likewise, a CPS may be unconnected with a SoS. The CPS, however, always contain embedded software, which may be connected by internet or non-internet technologies to a SoS. Lastly, the CPS should have a series of human–machine interfaces [67].

The nature of CPSoSs facilitates complying with the previous requirements. However, there are a few additional architectural requirements that we put forward, focusing on integration-oriented architectural components. Inspired from the functioning of smart grids as addressed by [110], we imply the following requirements.

**Multi-actor and multi-level:** Administrative authorities and decision-makers can interact within an enterprise’s CPSoS, each defining its objectives over an enterprise part. A typical enterprise’s CS (e.g., a smart pallet) interacts with other CSs (e.g., other smart pallets) and can also be included in larger parts of higher constituent bodies (e.g., a container or truck). Actors can be egocentric or altruistic and the system must fairly serve their interests. From a control point of view, this suggests dividing the system into several levels [110], each managed by one or more CSs. The CSs should then be integrated to ensure the enterprise’s coherency. This requirement mainly complements the managerial independence with situation awareness (i.e., part of a bigger whole) and associated business rules.

**Multi-objective:** Actors managing an enterprise may want to define additional objectives, which could intervene with the CPSoS. CSs must be able to adhere to (apparent) contradictory objectives, such as “increase resilience” and “reduce costs”. Possibly, these objectives can change over time and are omnipresent. Additional (conflicting) goals and constraints may appear as a CPSoS progresses, such as a vessel’s tardiness constraint being threatened by an overloaded truck’s capacity. We devote a special attention to the notion of resilience with respect to this requirement. Objectives can be expressed in terms of goal functions which could be specific resilience metrics in a CPSoS. The use of objective functions is an approach to assess the CPSoS functionality and capabilities in an attempt to control the resilience of an enterprise. Choosing and measuring some metrics for a specific context can lead to resilience gains. Such metrics may be qualitative or quantitative, external or internal, short-term or long-term focused, and can be tailored to specific resilience aspects (e.g., readiness, response, and recovery). Similar to the notion of emergent behaviors, objectives can be dynamic (e.g., appear and disappear, intensify and diminish, etc.) and may propagate further in a CPSoS.

**Flexible hybrid micro-macro integration:** The CPSoS must ensure a balance between omnipresence micro and macro objectives. Changing enterprise specifications and business rules on a rolling time horizon requires flexibility on balancing system objectives at run-time. That is, the CPSoS should have the capability to override macro-level objectives to micro-level objectives, and vice versa. Furthermore, as objectives may be non-deterministic and appear or disappear, and thus be temporal or partial of importance, the CPSoS should be hybrid of nature. Here, strategic, tactical, and operational decision support is needed. For example, a hybrid electric vehicle that is charging via a smart grid may decide to transfer energy to a household because of a sudden conventional refueling and energy price deviation.

**Meta-management feedback:** The resolution between micro- and macro-goals is a meta-management objective in itself, as it dictates the conflict-resolution strategy and hence the behavior of the system [110]. CSs should receive continuous feedback on their decisions, from various managerial perspectives (e.g., collaborative, competitive, etc.). Gorod et al. [75] also pointed out the importance of a feedback loop. The CPSoS should consider that each CS is a constellation of influences and how these influences are interpreted and applied.

Given this discussion on the requirements, we expect that no single CS design can fulfill all these requirements to its fullest extent, spanning the whole CPSoS. Similar to the functioning of smart grids [110], we require a mixed solution integrating several heterogeneous CSs. This imposes additional requirements:

**Interoperability:** CSs should be able to exchange states and data and interact following the business logic, as they interact through cyber or physical channels. The diversity of the CS parts, including legacy systems, leverages the SoS’s purpose [92]. Interoperability provides a basis for behavioral adaptability in terms of system emergence [80]. A specification of standard interfaces [74] and protocols should be properly defined. For example, technical, syntactic, semantic, and organizational interoperability are required [73]. This results in design rules and constraints. Infrastructure and data ecosystem initiatives such as Gaia-X [111] or the International Data Spaces (IDS) [112] may put forward this notion.

**Intelligence amplification:** Humans participating within and between humans and the CSs also impact the CPSoS. Several perspectives among participating parties must be entailed. Inherent to human’s involvement are the resulting emergent behaviors [78]. An intelligent agent (e.g., a piece of software) can emulate behaviors and interact with humans to upskill mutual decision-making capabilities on understanding emergent behaviors [19,20]. A CS can complement a human’s expertise but also vice versa, taking the best of both by working in harmony.

Last, when architecting a CPSoS there are some design principles that one should consider. Dahmann and Baldwin [66] elaborates on various SoS design types, which we modified to **CPSoS design principles** as follows.

- A *directed* CPSoS contains a coordinator that centrally controls all the CSs of a CPSoS. In such a system, the individual CSs maintain the ability to operate independently, but their operational mode is subordinated to the central managed purpose [66].
- A *virtual* CPSoS lacks a central authority and depends upon relatively invisible mechanisms to fulfill its purposes. In this design, there is no centrally agreed upon purpose for the CPSoSs, yet there is an invisible mechanism to maintain it.
- In a *collaborative* CPSoS there is a decentralized control. This means that the CSs collaborate to fulfill the agreed upon central purposes while there is no central coordinator enforcing power [66].
- An *acknowledged* CPSoS has clear objectives, management, and resources, but lacks control over the systems which they depend on to meet their objectives. Basically, an acknowledged (CP)SoS design falls between the directed and collaborative design. The SoS has objectives, management (e.g., authority), and resources for the SoS, while the CSs retain their independent ownership, management, and resources [66]. Acknowledged SoS are not typically new developments but overlays on existing or new systems, which were developed and are being used in different contexts. The objective can be to improve the way the CSs work together to meet a new need, involve new systems or technologies, or support (CP)SoS capability objectives to have application outside of the initial (CP)SoS [66]. An example is a governmental institute that aims to stimulate industries to become more environmental friendly. Such an institute typically has objectives, management, and resources, but no control of the systems that actually execute the actions.

## 5. Reference Architecture Design

Based on the functionality and underlying requirements we formulated in the previous sections, in this section we design an EA model that formally specifies our proposal for a reference architecture for detection and monitoring of emergent behaviors and resilience “by design”. One of the main distinctions between our reference model and the existing models is that we focus on the detecting and monitoring of emergence, from a functional architectural perspective. While languages such as SysML, CML, and UML are commonly used when specifying and designing SoS architectures, we decided to use ArchiMate [13] for modeling the EA, as our main focus is not per se on system design, but rather on capturing emergent behaviors and their management mechanisms in the broader organizational context of business operations and process execution. We aim to support modeling, reasoning, and model-based (quantitative) analysis of the functional components in an architectural blueprint. To this end, the ArchiMate language provides a rich modeling expression, ranging from motivation aspects (such as requirements and goals) to system design aspects (such as application landscapes and their corresponding IT and physical infrastructure). Therefore, ArchiMate models provide anchors to other domain specific languages (e.g., BPMN, UML, SysML, etc.), that might be needed for a possible further refinement of some parts of the proposed architecture and for future extensions and derived instantiations of the reference architecture. ArchiMate is also a commonly used modeling language and framework and perceived as the de facto standard for EA modeling [113].

**Figure 3:**
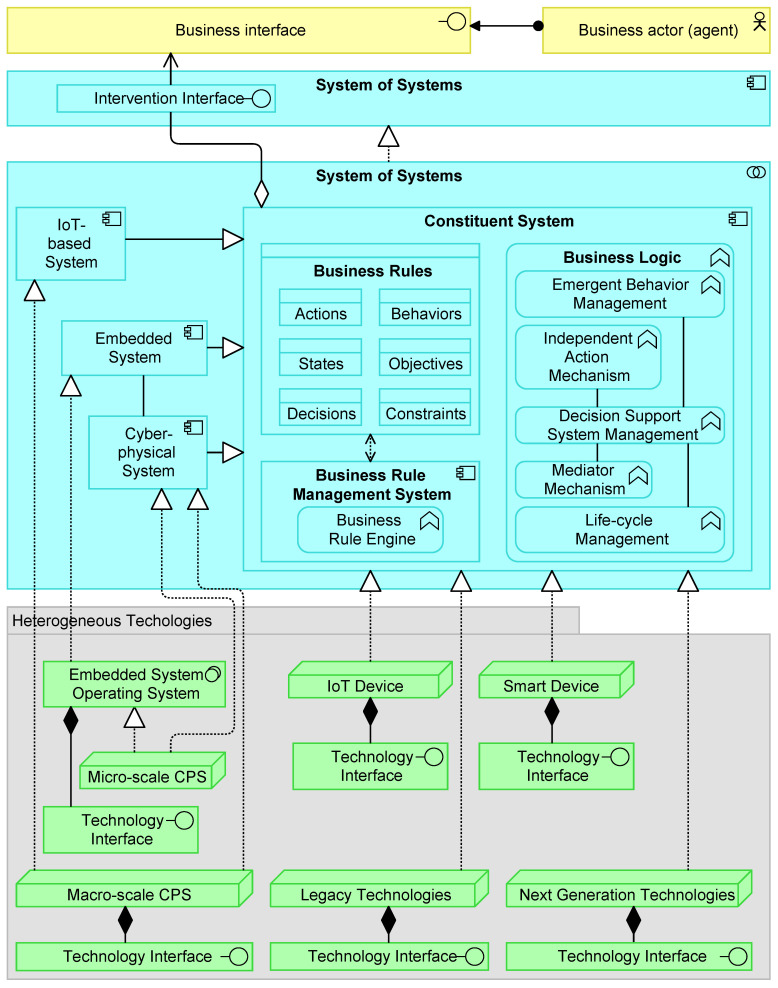
Reference enterprise architecture.

Figure 3 proposes an EA based on the positioned requirements. The EA shows four layers as well as the relationships within and across the layers. For building this architecture, we took some inspiration from references found in our literature study, among others [19,20,69,96], and a tertiary study on architecting SoS [84]. The architecture is proposed based on group discussions among the authors and project members, involving among others IT consultants, enterprise architects, computer engineers, and researchers. Besides that, we reviewed excerpts of the EA based on discussions with colleagues that have experience with EA modeling and are not directly involved in this paper. The intention was to compose the architecture such that it is sufficiently generic to be adapted to various domains. The architecture is realized in multiple iterations of which we discuss the final one as shown in Figure 3. Below, we first elaborate on architectural components regarding the functional requirements. Last, we provide a motivational model that links the design goals and obtained requirements to corresponding architectural building blocks.

### 5.1. Constituent System

A Constituent System can work collaboratively with other Constituent Systems to realize the behavior of the System of Systems application. The Constituent System application component dictates that a CS can operate independently, even if the CS is not part of the SoS. However, for a CS to be part of a collective of CSs, and thus a SoS, the System of Systems application component represents an entity that plays a critical role in the creation, achievement, sustenance, or operation of the SoS. Further, a CS can operate independently, and, for example, be dynamically part of a SoS comprising of other CSs. This clarifies the SoS *evolutionary development*, *managerial independence*, and *operational independence* prerequisite as well as some of the SoS design principles.

Via an Intervention Interface, an agent (e.g., human) can interact with a CS, and vice versa. However, not all CSs are able or aim to interact with an agent (via the Business Interface). Similarly, not all Business Actors are able to interact with certain CSs. In turn, the interactions across this interface may also affect the Business Rule (e.g., adding human’s business rules or meta-feedback). Through the use of interfaces, the architecture supports different specifications of behavior (or contract) that CSs and business actors agree to meet. The Intervention Interface also justifies the *intelligence amplification* (see also Section 5.4) and *interoperability* requirement.

The exemplified variety in technology components that realize a CS illustrate a *heterogeneous* applicability, including Legacy Technologies and Next-Generation Technologies. The specialization components belonging to the shown application components IoT-based System, Embedded System, and Cyber-physical System illustrate some elements that are a particular kind of a Constituent System. For example, some CS functionality may be incorporated only to a limit extent, or not at all, under resource-constrained IoT-devices. Likewise, the corresponding Business Rules may differ. To exemplify which business rules can be covered, we included Actions, Behaviors, States, Objectives, Decisions, and Constraints. However, it would go beyond the scope of this paper to address any data specification of a business rule. Noteworthy to mention is the Business Rule Engine as part of the Business Rule Management System facilitates the execution and management of business rules in a runtime environment. The Business Rule Engine makes also sure that there is a proper alignment (*interoperability*) between cyber and physical channels.

### 5.2. Business Logic

**Figure 4:**
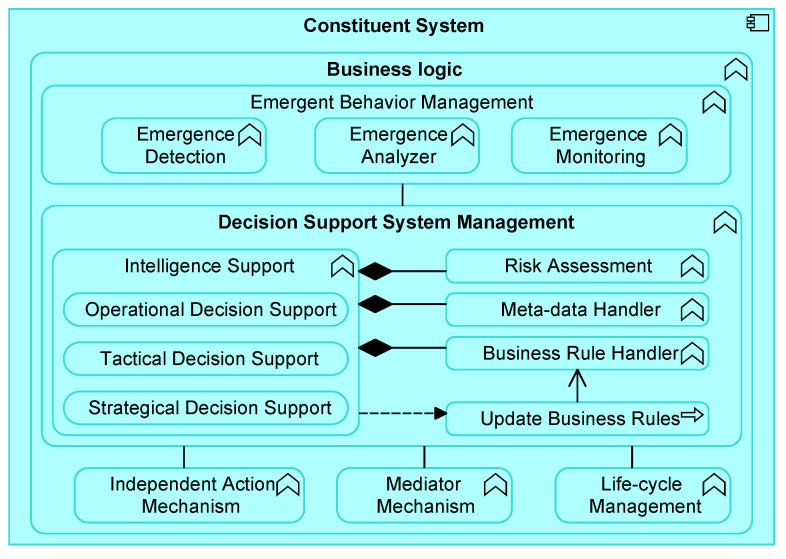
Architectural view on the business logic component.

Figure 4 shows the Business Logic component in more detail. A CS has its own Business Logic component, consisting of several application functions. This component is led by a Decision Support System Management functionality, which is capable of operational, tactical, and strategical decision-making based on a collection of functional modules (e.g., Risk Assessment, Meta-Data Handler, etc.). The Independent Action Mechanism comprises *interoperability* aspects by also enabling decision-making without, for instance, an *Intelligence Support* functionality on board. This may include legacy systems or safety critical systems which are supported by this means. The Emergent Behavior Management functionality contains capabilities related to a MAPE-cycle. While the monitor and analyze application functions are captured within the Emergent Behavior Management, the plan and execution part are part of the Decision Support System Management because this component can also consider other analytics methods to accommodate.

### 5.3. Cyber-Physical Capabilities

The physical and computational components of a CPS are integrated at the Cyber-physical System application component. This component is a specialization of a CS. Because of being a specialization, similar properties of a CS are applicable, such as that the associated CS is not necessarily part of a SoS. Thus, the CPS may also be unconnected with a SoS and act as an autonomous subsystem.

The Cyber-Physical System application component is realized by a CPS technology node. The Micro-scale CPS and Macro-scale CPS nodes represent a computational or physical resource that hosts, manipulates, or interacts with other computational or physical resources. A Micro-scale CPS can be a single physical node (e.g., sensor and actuator), while a Macro-scale CPS covers multiple physical nodes.

In essence there is no difference between technology components that realize a Constituent System application component directly (e.g., the depicted IoT-device) and technology components that realize an intermediate application component (e.g., the depicted Macro-scale CPS). The latter one is a specialization of a Constituent System, while a directly realizing technology component has no intermediate application component. Such an intermediate application is used to exemplify some conventions of what a Constituent System can comprise. For example, it shows that a Constituent System can be realized by multiple technological sources (e.g., sensors and actuators) which collectively shape the creation, achievement, sustenance, or operation of it. Notice that the shown application components (except the Constituent System) and technology components are just an exemplification of the variety of components. In practice, these components can comprise omnipresent and heterogeneous applications and technologies that can be defined as a Constituent System. What is considered as a Constituent System depends on the system design (see Section 5.5) and how fine-grained the Business Logic and Business Rules are implemented.

The Constituent System can also assimilate recorded physical data in an enriched manner. Data enrichment may comprise merging data from external sources (e.g., the internet), which are not always completely controlled or understood. A technology node, such as an IoT Device, can parse information about physical processes, which can be embedded by the Constituent System. To this end, in principle, a Constituent System can comprise physical processes up to the atomic level. However, technological constraints (e.g., computational limitations) may limit the encapsulation of such processes within the application functions of the Constituent System. For example, it may not be worthwhile for typical IoT devices to embed sophisticated Decision Support System Management functionality within the business logic, given that these devices often have limited battery and computational power. Some technological components may also hide (parts of their) business logic or may not be accessible (e.g., due to legacy reasons). Besides that, consider the notion that even simple devices can already have almost a dozen operations, several communication protocols, and various communication interfaces. For these reasons, a Technology Interface makes sure that the technology services offered by a node can be accessed and are part of the Constituent System.

Notice that the computational or physical resources (e.g., the IoT Device component) are ultimately responsible for hosting, manipulating, or interacting with other computational or physical resources. The technology components may also accommodate computational resources externally or within a resource-shared infrastructure, such as in the cloud. This also means that a technology component can represent an artificial node that only acts as a hosting, manipulating, or interacting computational or physical resource. In a similar fashion, one technology component can also realize multiple CSs, because a component may differentiate between computational or physical resources itself. For example, one physical Smart Device can have two dedicated compartments, which are separated as technology components (i.e., two CSs that do not necessarily belong to one SoS). The modeling of the physical layer is left for further research.

### 5.4. Intelligence Amplification

The concept of *intelligence amplification* can also be positioned within the proposed EA. A human entity can interact with a CS such that both the human and the CS can complement each other to enhance human decision-making. The interfaces make it possible for humans to provide input and feedback to the CSs in such as way which can be handled by them. Likewise, a CS may have the capability to understand and reason about the human’s input. One CS may be solely dedicated to interacting with a human and thereby it can act as a bridge between other CSs. However, a human may also communicate with multiple CSs directly.

### 5.5. System of Systems Design Principles

It is worth mentioning that the proposed reference architecture embeds the four SoS design types as mentioned by Dahmann and Baldwin [66], namely, directed, virtual, collaborative, and acknowledged. These design principles constitute many of the requirements, such as *operational independence*, *managerial independence*, *multi-actor and multi-level*, *multi-objective*, and *flexible hybrid micro-macro integration*.

**Directed System of Systems**: The System of Systems application collaboration component of Figure 3 aggregates Constituent System application components that may cooperate to perform some task. The Constituent System component is assigned to a Business Logic application function, which can comprise a Mediator Mechanism as part of the Decision Support System Management. As further specified in Figure 4, the Intelligence Support function as part of the Decision Support System Management can make operational, tactical, or strategical decisions. For example, about how to collaborate with other CSs on the long term. In turn, these decisions are concretized via business rules, for instance, in the form of agreements. A Constituent System application component can act as a central authority controlling what other CSs should do, via propagation of the business rules. Notice further that one Constituent System application component can be part of more System of Systems application collaboration components.

**Virtual System of Systems**: The reference architecture allows CSs to be part of a SoS, while there is no agreed purpose. Emergent behaviors may arise, but the SoS relies on concealed mechanisms to perform its overall goals. Basically, then, the CSs do not know about each other. As a SoS progresses, some of these invisibilities may become discovered via the Emergent Behavior Management, after which the system might evolve. However, this is not assumed to be the case in a completely virtual SoS.

**Collaborative System of Systems**: Agreements about standards and how to maintain them may be originated centrally, but the CSs can start their own (unplanned) collaborative mechanisms. Therefore, CSs can voluntarily work together to address common or shared interest. An example of this design type is the Internet in which one organization is taking care of standards and communication protocols and participants can start their own collaborations (e.g., a local internet).

**Acknowledged System of Systems**: An acknowledged SoS tends to be common in cases with top-level objectives balanced with the objectives of the owners of the systems which support the SoS [66]. The SoS control unit does typically not control the CSs in the SoS but serves an influencing position rather than directing the CSs to meet SoS needs. Thus, unlike directed SoS, CSs in an acknowledged SoS can work freely without depending on the system. Consequently, changes in the systems are made based on collaboration between the SoS and the system and not based on pure top-down authority from a SoS manager or CS’ emergent behaviors.

#### General System of Systems Design Principles

The *multi-actor and multi-level*, *multi-objective*, and *flexible hybrid micro-macro integration* prerequisites are intrinsically embedded in the Business Rules and Business Logic components and can provide a new context (e.g., decision authority, motivation, action, legacy, etc.) for fulfilling a (CP)SoS’s purpose. A typical SoS evolves of a set of existing and new systems, which can become components of a larger SoS while preserving autonomy as individual systems [66]. This intrinsic property constituted within the business rules and business logic component enables, among others, an *evolutionary development*.

Building further on this notion, the proposed EA allows a dynamically adapting CPSoS in which the CSs can be at different evolutionary states and run- and design times. For example, CSs can interact with older versions of themselves or of other CSs or SoSs. The depicted EA of Figure 3 also allows the incorporation of the four CPSoS design principles coherently. For example, as a SoS progresses (e.g., in time), new collaborations, such as alliances, may be shaped. These collaborations can be temporary of nature or subject to change after which a new system design can emerge. Moreover, multiple nested CPSoS design principles may be exhibited in a CPSoS. For example, a fleet of barges may be centrally controlled, but one barge within that fleet can subordinate a collaborative CPSoS design principle to execute its daily operations.

Furthermore, these new contexts on top of the aforementioned functionality, do not only foster uniting organizational-wide behavior and goals with the behavior of local autonomous entities, but also allow nested CS and SoS design patterns. A nested design pattern can be described as a CS that contains a SoS in itself. A CS may not (yet) be aware of a nested SoS inside the CS. We can already say that a CS has nested SoS properties if there is or was at least one CS within it.

### 5.6. Architectural Motivation Model

As additional part of the validation of the EA model, we describe a so-called EA motivation model which shows the motivations and reasons that guided the design of the proposed EA. Alignment and traceability between requirements and the architectural elements is of importance, because they shape the overall architectural design [114]. By linking the goals with the discussed requirements and, consequently, the requirements with the architectural components, we aim to demonstrate the cohesion of the EA as well as how the goal of achieving enterprise resilience could be realized.

**Figure 5:**
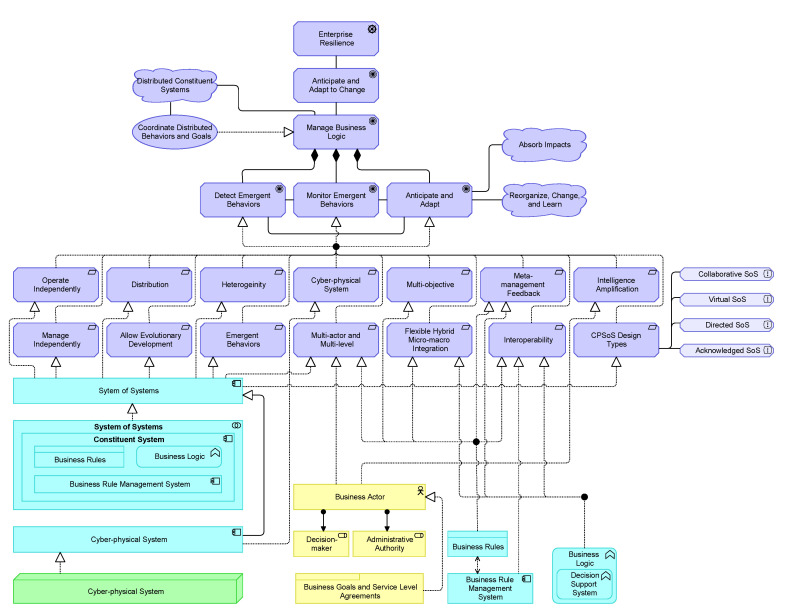
Motivation model linking the design goals and requirements to the corresponding architectural building blocks.

The Open Group proposed an extension to the ArchiMate language [114], which we used to include requirement management aspects in the context of the proposed EA. High level strategies and goals of an organization (e.g., obtaining a resilient enterprise) could then be linked to its architectural components [115]. After considering the goal motives of this study, the formulated requirements, and architectural components, we composed the motivational EA model as depicted in Figure 5. This model is made based on an assessment that has been done after the reference EA (Figure 3) was proposed. By doing so, we perform an additional validation step which goes beyond the proof-of-concept implementation of Section 6, albeit is confined to the limits of this study’s purpose. For a more detailed explanation of the ArchiMate language notation and concepts we refer the reader to the work in [13].

The main driver of the motivational model is to achieve a resilient enterprise. Following the proposed definition as given in Section 3.3, resilience focuses on anticipating and adapting to change using the inherent CPSoS capabilities. We positioned this as a goal, which is addressed by narrowing down to the management of business logic. The notion of business logic should be interpreted while keeping the context of CPSoS in mind, considering, for example, the coordination of distributed behaviors and goals of the CSs. Under the business logic management goal demarcation, we confine to detecting and monitoring of emergent behaviors, and the anticipating and adaptation of the CPSoS’s business logic. The latter one is derived based on the CPSoS definition.

The discussed requirements of Section 4 are explicitly addressed in requirement components. Our hypothesis is that taken together these requirements realize the goal components. In turn, each requirement component is related to core architecture elements that realize them, such as processes and applications. Please notice that the depicted core components are illustrative of nature and sketching how they can be related to the requirements. We are aware that the mapping of the requirements mentioned in Section 4.3 to the reference EA and EA motivation model may not be fully self-explanatory (and complete) in terms of reasoning on each relationship. However, an important observation lies in the fact that each requirement can be facilitated by the reference EA. Further investigation is needed to determine to what degree a requirement is satisfying an architectural component.

## 6. Proof-of-Concept Implementation

The proof-of-concept application in this paper focuses on examining the effectiveness of some contributions of the presented architecture. We consider a multimodal logistics case study, involving no central governance, no unified design, no common goal, and scarce resources for which autonomous vehicles need to compete. The presented case study comprehends an instantiation of the presented EA. From hereon, we refer to this case study as MultimodalLoCo.

### 6.1. MultimodalLoCo Logistics Case Study Description

**Figure 6:**
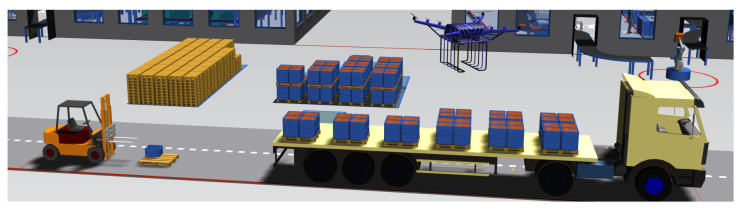
MultimodalLoCo case study. Inspired by Bemthuis et al. [20] and modified in Tecnomatix Plant Simulation v14.

The case study concerns transport of fresh and frozen food products of which the quality deteriorates over time. More specifically, we build further on the work of Bemthuis et al. [20] in which a fleet of heterogeneous cargo transporters moves pallets from one place to the other. The study concerns different modes of transport which partially rely upon the behaviors, actions, and performances of other transporters and the overall system. Please see Figure 6 for a visual impression about the case study. Multimodal transport refers in our case study to the transportation of logistics cargo under a single contract, but performed with at least two modes of transport. A single enterprise orchestrator is overseeing the shipments and is responsible for the overall performance, but the autonomy of the cargo transporters is not under this command. We refer the reader to the work of Bemthuis et al. [20] for more details about the case study.

This multimodal logistics case study is suitable for validation of the architectural components, because emergence in this context includes a wide range of (global) effects occurring unexpectedly, and sometimes without apparent reasons. For example, delays and deadlocks (e.g., traffic jam) could occur, but also desirable behaviors such as synchronicity, stability, or coherence. Furthermore, the case study concerns a variety of independent transport entities, which have their own autonomy, and computational and physical elements. Moreover, this case study, involving the logistics domain, is suitable because logistics supply chains are becoming progressively less centralized, imposing a form of distributed autonomous business logic, and some central control mechanism (e.g., 4PLs and sub-contracted transportation service providers). In a broader sense, this is in line with how contemporary organizations operate. Organizations are typically characterized by a multitude of heterogeneous (local) actors that pursue their own, sometimes conflicting, goals, norms, and values [116]. This makes the considered multimodal case study involving the transportation of goods under the presence of an enterprise orchestrator a fruitful source to explore.

To be able to operate appropriately under disruptive events, MultimodalLoCo requires capacity for resistance and the capacity for recovery: resistance in the sense of reducing the impact of disruption, and recovery to quickly resume normal operations after a disruption. MultimodalLoCo aims to become a resilient enterprise by overcoming potential disruptive events. More specifically, MultimodalLoCo would like to get insights into how capable their organizational structure is in anticipation of and during times of disruptive events.

### 6.2. Model Motivation

Similar to Bemthuis et al. [20], we decided to model the case study using agent-based modeling. Our literature review (see Section 4) already indicated that this is an often used approach in modeling SoS and emergence. Agent-based modeling concerns the modeling of distributed intelligent systems composed of agents that cooperate and coordinate to solve their local goals and global goals [117]. This modeling technique is suitable for analyzing such autonomous and distributed systems, because it can capture emergence and it provides a natural description of a SoS system [78]. A study of Singh et al. [118] already showed that agent-based systems is an effective way to explore emergent behaviors. In particular, we use an agent-based simulation model for understanding the emergent behaviors. This type of simulation is useful because of its ability to represent several behaviors (e.g., from humans or spatial environments such as CPSs) realistically, accounting for bounded rationality, heterogeneity, interactions, evolutionary learning, and out-of equilibrium dynamics [119].

### 6.3. Model Description

**Figure 7:**
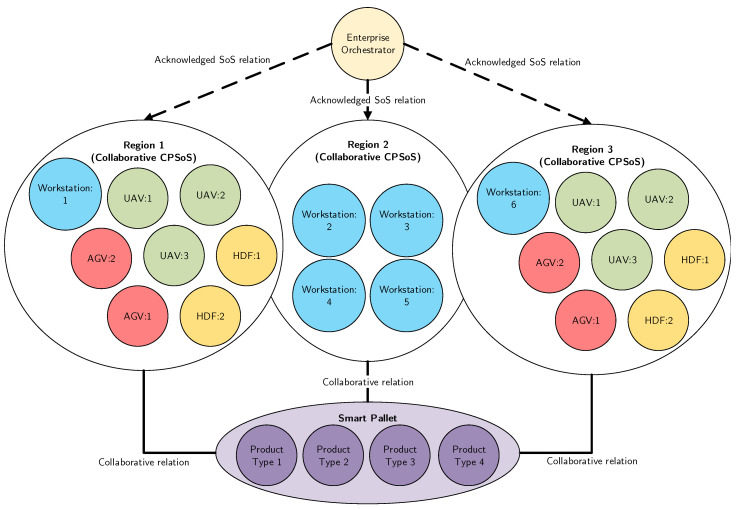
Conceptual framework of the case study’s constituent systems and CPSoS design relations. Each colored part depicts a constituent system. The smart pallet constituent system contains one of the products, which is considered as a nested constituent system.

Operationally, MultimodalLoCo is a CPSoS spanning multiple CSs. We categorized the following main entities in this case study; (i) workstations, (ii) smart pallets, (iii), cargo, (iv) transporters, and (v) enterprise orchestrator. Within these categories, there are multiple distinctions one can make. After discussions with the consortium partners about what should be defined as CSs and how these are related to each other, we constructed the conceptual mapping of the CPSoS design principles and CSs specifications as shown in Figure 7. Each colored circle or eclipse represents a CS which is geographically distributed and managed independently.

**Figure 8:**
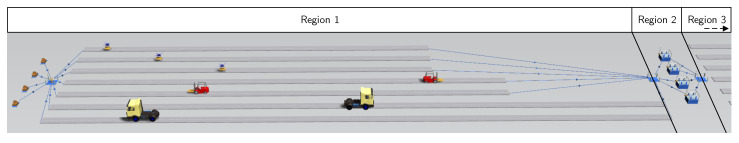
A graphical representation of the simulation model. A product flows from region 1 to region 2 after which it undergoes processing. After a product is processed, a transporter moves the cargo from region 2 to region 3. For representation purposes, region 3 is left out of this illustration, but this region is comparable to region 1 (see [20] for more graphical representations).

The case study involves three designated regions in which operations take place (see Figure 8). In region 1, there are transport requests initiated by a workstation and carried out by the transport units. A transport request involves moving a pallet from the workstation in region 1 to the workstation in region 2. This pallet contains one of the products. Region 2 processes the products. After that, the smart pallet can be picked up and transported from a workstation (in region 2) to the final workstation in region 3. This transport is again carried out by independent operating transporters. Figure 8 gives an graphical representation of the case study as implemented in the agent-based model.

There is one central enterprise orchestrator (a human entity), which is acknowledged by the CPSoSs operating in the above mentioned regions. The role of this orchestrator is to stimulate and give incentives for the transporters to carry out their tasks efficiently. In all regions, we consider a collaborative CPSoS design in which there is an agreed upon purpose to fulfill the tasks, yet each transporter can make its own decisions. The transporter CSs compete with each other about the assignment of transport tasks, by means of business rules.

**Figure 9:**
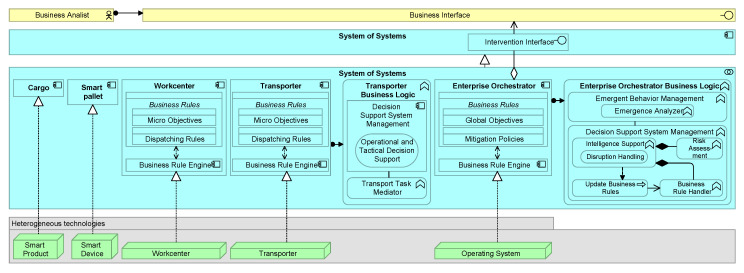
MultimodalLoCo’s enterprise architecture.

Figure 9 shows how we instantiated the proposed reference EA for this use case. Each of the categorized entities is considered as one CS within this representation, but varying levels of details are given. The previously mentioned main entities are exhibited as *Constituent Systems*. The *Workcenters*, *Transporters*, and *Enterprise Orchestrator* have their own set of business rules and a business rule engine that makes sure that the business rules are appropriately managed and executed. The business rules within the *Workcenter* CSs involve workcenter-initiated dispatching rules (see Section 6.4) and the *Transporter* CSs’ business rules involve vehicle-initiated dispatching rules. As part of the enterprise, the performance achieved within the *Workcenter* and *Transporter* CSs are considered as local objectives, while the *Enterprise Orchestrator* CS considers global objectives.

We exemplified two business logic components in Figure 9. The *Transporter Business Logic* concerns operational decision support, which takes care of the dispatching rules handling. The *Enterprise Orchestrator* focuses on (disruption) mitigation strategies. These strategies are captured in business rules, which are produced based on the *Enterprise Orchestrator Business Logic*. More precisely, this business logic involves analyzing the behavior that emerges from the interacting CSs. A human (*Business Analyst*) assesses performance expressed in terms of realized product outcomes (e.g., product cycle time, decay level, etc.) when a product leaves the system. The human considers the emergent behaviors and decides what actions to undertake and what new or updated business rules should be invoked.

### 6.4. Experimental Set-Up

In our previous work [20], we focused on minimizing the cargo’s quality decay against minimizing the average cycle time per product. Here, we build further on this work by also considering (i) sources of disruptions, (ii) disruption mitigation strategies, and (iii) local against global objectives. Similarly to the work in [20], we also focus on dispatching rules as primary business rules. For our purpose, we consider only one of the scenarios and experimental settings, namely, the configuration with the highest accumulated counted ranking of the average end decay level (higher is better) and the average cycle time per product (lower is better). It appears that there are two best settings (scenario 2 with RS configuration and scenario 2 with HS configuration). We decided to go for the first one, which considers 6 UAVs, 4 HDFs, and 4 AGVs, and as product-initiated dispatching rule (i.e., business rule) *shortest travel distance* and as vehicle-initiated dispatching rule *random*. The chosen transport modalities are also represented in Figure 7 and Figure 8.

Let us give a summary of the considered simulation model parameters, based on the work of Bemthuis et al. [20]. The three vehicle types each have a different speed and capacity. The distance a vehicle needs to travel is also dependent on the vehicle type (e.g., the lane length differs). In total, there are four product types that each has a different size and undergoes a different quality decay process. Regarding the latter one, a product has a specific quality decay function which depends on the mode of transport. The size properties impose restrictions on the task allocation process, such as that a large product may not be transported by a (small) UAV. An additional constraint is given that products of type 2 may not be combined with products of type 3 in one trip.

The implementation of the business logic (see also the logic flowcharts in [20]) and the use of business rules allow the process to arise some form of emergent behavior from local interactions between parts of the initially disordered system. In fact, the dispatching rules may be initiated at unpredictable moments, especially when a disruption disorders the state of the enterprise. In this context, we consider resilience as the degree to which the enterprise has the resources and is capable of self-organizing with regard to potential disruptions. The enterprise is denoted by the enterprise orchestrator agent, which is responsible for the quality of the end product as well as the overall enterprise efficiency. We express metrics of enterprise resilience as two key performance indicators (KPIs): the *end decay level* and the *cycle time* of a product. These two KPIs represent a global objective and indicate how resilient the obtained overall coherency as denoted by the products leaving the system is (e.g., as delivered to customers or third parties).

### 6.5. Results of Analysis

The goal we had in mind when proposing this architectural design was the realization of a symbiotic relationship between the autonomy of distributed business logic on the one hand, and a central control on the other hand. To illustrate this, we discuss some experimental results on (i) the impact of disruptions, (ii) disruptive mitigation strategies, and (iii) macro- versus micro-objectives.

**Figure 10:**
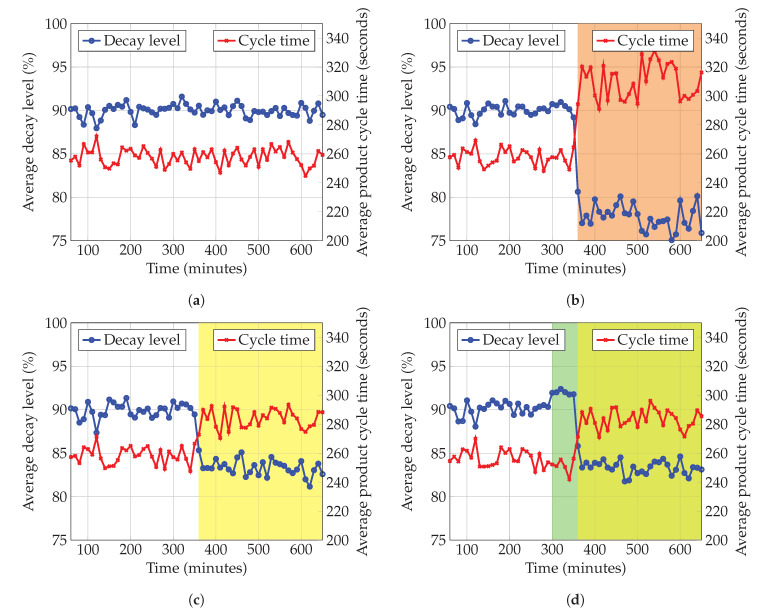
The impact of disruptions and disruption mitigation strategies. (**a**) No disruption. (**b**) With disruption after 6 h and a reactive mitigation strategy. (**c**) With disruption after 6 h and an unchanged strategy. (**d**) With disruption after 6 h and a proactive mitigation strategy.

Figure 10 presents some experiment results focusing on (i) and (ii). The graphs display the *average product end decay* (in %) and *average cycle time* (in minutes) of a single simulation run for four disruption mitigation scenarios. The numbers depict average outcomes based on realized products (e.g., products that leave the system) within a 10 min interval. The first hour is neglected, due to a warm-up period. Below, we discuss these results.

Figure 10a shows the normal behavior, without an expected (detrimental) disruption. As we can observe, the results tend to become reasonably stable after a couple of hours. In the second scenario (Figure 10b), we invoked a disruption after 6 h. The disruption entails a travel speed decrease of all automated guided vehicles by 75%. The results indicate that both performance indicators are substantially impacted. The product decay level dropped from roughly 95% to 78% and the average cycle time of a product increased from approximately 260 to 310 s. As a third scenario (Figure 10c), we build further on the second scenario and consider the mitigation strategy of increasing the speed of the other transport modes by 33% at the moment of disruption. Here, the disruption has been partially repelled. Nevertheless, in comparison to Figure 10b, the impact can still be considered as disruptive. In this scenario, the decay level dropped to 83% and the cycle time increased to roughly 290 min. We further notice that the impact on the KPIs depicted in Figure 10b,c are both affected in an almost similar fashion, except that the scale of impact is larger for the unchanged mitigation strategy. This can partly be explained by the use of the same random seed values in the simulation model. Figure 10d goes one step further, by already using the mitigation strategy one hour before the disruption will occur. We just assumed that there is a certain emergence detection mechanisms, which was capable to proactively detect that a disruption will occur. The results indicate that disruptions and mitigation strategies yield various emergent behaviors.

**Figure 11:**
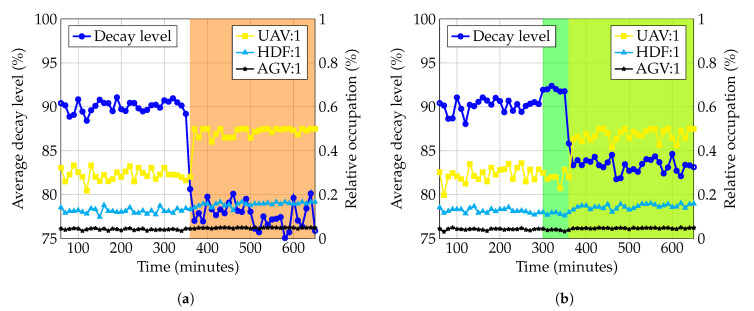
Macro- against micro-goals in disruptive scenarios. (**a**) With disruption after 6 h and an unchanged strategy. (**b**) With disruption after 6 h and a proactive mitigation strategy.

To examine how macro- and micro-goals are intertwined, we analyzed how the *average vehicle occupation* (as micro-objective) behaves against the *average product end decay level* (as macro-objective). A micro-objective is applicable to individual CSs, while the macro-objective covers a resultant end value which can be used to assess the emergent behaviors of interacting CSs. The latter one can be considered as being part of the enterprise regulator agent. Figure 11 shows the *average occupation rate* of one vehicle of each type. In both depicted scenarios, it seems that most occupation rates increase but the final decay level is still drastically affected. Furthermore, when comparing Figure 11a with Figure 11b, it appears that the disruptive impact on the global scale is considerably absorbed with the proactive strategy.

Although preliminary, this analysis sheds some light into how resilient an enterprise can be under disruptive scenarios. Let us briefly discuss some inferences we can obtain from the analysis. In the view of emergent behaviors, we observed that the various CSs work together, cause emergence (as expressed with a realized KPI), and achieve a “higher” goal by interacting. In the light of enterprise resilience, the presented scenarios indicate what emergent behaviors are realized by the system. The illustrative scenarios suggest that we can define actions (e.g., change in business logic) that affect a particular emergent behavior. This can imply that the CSs within the CPSoS can reorganize, change, and learn using capabilities to absorb the impact. Notice that we give limited attention to applications that manage the disruption effectively and timely. Instead, we only showed how one could possibly deal with a disruption. Assessment of effective emergence control strategies, including self-learning capabilities, is needed in future research.

## 7. Discussion

In this section, we discuss potential threats to validity of this study and how we mitigated these threats. We address threats in the context of the SLR, requirement proposition, architecture proposal, and case study.

### 7.1. Systematic Literature Review

Internal and external dimensions of the validity of the SLR [23,24] were considered to minimize researchers’ bias and to ensure the reliability of this review. The internal dimension concerns how well the study answers the research questions and the bias degree of the study, while the external dimension relates to the generalizability of the study’s findings.

This study uses a SLR protocol based on well-known guidelines and recommendations of Kitchenham [23], Kitchenham et al. [24], and Rouhani et al. [25]. The use of such a protocol strengthened our internal validity, because it exists of verifiable procedures. The search strategy was designed to mitigate selection bias. The search term and inclusion and exclusion criteria were refined before the execution of the search, thereby providing creditability of ensuring a comprehensive and unbiased selection process. The search strategy was proposed by the principal author and refined based on reviews from the other authors.

In order to mitigate the potential threat of including a limited selection of digital libraries and ineffective search strings, we initially performed a sample search on one search engine and discussed a selection of initial papers to confirm the potential literature base. After that, we refined the search string and performed the search in three commonly used digital libraries in scientific research. Although the limited selection of three search engines may be a threat to validity, we believe that most of the relevant literature is captured, because there were already quite some literature duplicates. Besides that, some additional papers were added after a manual search by the co-authors of this study. Nevertheless, our review protocol may be used to extend the results by using more databases or (reverse) literature snowballing techniques.

Another threat exists in the composition of the search string. CPSoSs cover a wide range of aspects and application domains. Furthermore, within the SoS field, there is an absence of standardized definitions for the concept [84]. Besides that, emergence is a relatively new field of study and no widespread, widely accepted, approaches to deal with emergence in SoSs are present in the literature [74]. Systematic literature studies addressing (CP)SoS or emergence are still rare. Even though our study brings together multiple fields in an attempt to unify some common principles, the results of this study may be tempered by a lack of standard terminology on the subject. For example, the inclusion of the term emergence may have excluded papers that address similar underlying concepts but that do not explicitly use affiliated terms. Furthermore, the inclusion of the constructs industry 4.0 and Internet of Things may have resulted in a larger body of literature, focusing less on this paper’s key concepts. However, we wanted to also include contributions that put less emphasis on the exact terminologies used in the other two parts of the search string but that focus on similar underlying concepts. Especially because Industry 4.0 is expected to reshape organizations such that CPS capabilities are needed [17]. These important concerns of this study are also addressed by the succeeded literature filtering process.

Last, the authors’ bias in the two screening steps could lead to unreliable results. To minimize the bias in the title, abstract, and keywords screening, two authors performed the initial screening. Disparities regarding the defined criteria were discussed and if no consensus was reached, the paper went through the next screening phase for further examination. In the case one of the authors was not sure about whether to reject the paper, the decision was made to not yet reject the article. By not yet rejecting the paper, we limit the exclusion of papers that may be of importance. Selection bias in the full-text screening was limited by first excluding papers based on their relevance regarding the defined criteria (in a similar manner as done with the title, abstract, and keywords screening) and then rating the studies based on two criteria. Although this may represent a concern of this study’s validity, we reduced the selection bias by including two authors rating the studies independently. The used scoring threshold is another concern to the validity, which may have excluded relevant papers. The authors may have had to make inferences about some studies, resulting in conceivable classification failures. However, we aimed to mitigate this risk by having two authors assessing the papers independently, comparing and discussing their findings, and consulting a third author in the case no consensus was reached. Nevertheless, the chosen absolute threshold value may have excluded papers which discuss some relevant aspects (e.g., propose a partial architecture). However, we aimed to base the reference architecture on mature research of papers addressing the two distinct criteria to a certain minimum level. In subsequent research, our research protocol can be used to extend the results by using a more rigorous selection process. In spite of the aforementioned potential threats, our literature overview contributes to synthesizing existing research.

### 7.2. Requirement Proposition

The requirement elicitation served as a foundation for the reference architecture development. The evidence obtained and evaluated by our SLR may be subject to validation concerns. To limit threats to validity, we considered a number of elicitation techniques to gather requirements. By the conducted SLR and additional discussions with project partners, we consolidated the literature base to requirements. Project partners were involved in brainstorming and prototyping sessions as well in the realization of the case study. Some of the project partners were experienced with the constructs, because of their previous case studies within a similar realm (see, e.g., in [19,20]).

Nevertheless, we may have missed some requirements, or some requirements may have been aggregated or defined differently when using other requirement elicitation techniques or under project setting conditions. To make sure that there was a common vocabulary during the project meetings, the principal researcher chaired the session and provided an introduction of the terms and gave a concise rehearsal of previous meetings. Furthermore, the consortium may have struggled with determining when they are done with requirement elicitation. This factor and other factors that could impede completion of elicitation activities were mitigated by (1) an incremental requirement elicitation approach (e.g., several meetings spread within a period of time, driven by information obtained), (2) building and documenting (visual) models (e.g., showcasing and walking through the graphical simulation models), (3) building further on case studies in which some project partners were already involved in earlier, and (4) periodically inviting external members to the meetings, which can bring in other topics of discussion. Besides that, some external researchers assessed the requirements and provided feedback, which is incorporated.

### 7.3. Architecture Proposal

The objective of the proposed architecture was to extend the known (CP)SoS models with a special notion on detecting and monitoring of emergence. The reference EA can help organizations with the adaptation of existing architectures, ICT infrastructures, processes, and relationships to support the transformation to become more resilient against (disruptive) emergent behaviors. Please note that the proposed EA is only a representation of the distilled requirements into a blueprint of architectural components and their relations, which imposes several threats to this study’s validity. For the realization of the EA, we mitigate the authors’ bias by using similar techniques as done by the requirement elicitation phase (see Section 7.3).

One of the strengths of the EA approach is that it has defined concepts and instruments to enable the modeling of abstract to concrete concepts, such as complex systems. While there are other languages such as UML, SysML [120], or CML intended to also enable modeling and specifying of CPS and SoS, we decided to opt for ArchiMate. One of the key arguments was that ArchiMate can be combined with other EA models and that it is an expressive modeling language. Future work can govern extensions or adaptations of the proposed EA, such as encompassing self- and time-awareness to support partial control of emergent behaviors. Such self-aware architectures are actively participating in the evolution and adaptation of the system [103]. Our suggestions for further research are in line with findings from the literate study of Cadavid et al. [84], which pointed out that the evaluation, implementation, and evolution of SoS architectures are underrepresented and require further research. Further research is also required on architecture modeling languages, e.g., on addressing self- and time-aware architectures, which can evolve over time.

Another point of discussion concerning the validity of the EA architecture, is the focus on describing the functional level of an architecture instead of how it can exactly be implemented. The reality is that in most enterprise CPSoSs, there is a complex goal and organizational structure, while operational performance must still be achieved under disruptive behaviors. Whereas the EA is about describing and linking components and interfaces, this kind of representations might have limitations in terms of capturing the complex dynamics in the development and management of a socio-technical system of heterogeneous systems. Providing more contextual background would be effective for expressing the benefits of using the EA as a source of reference. However, this entails a risk of misguiding efforts to make sense and appropriate this study’s goal. A similar reasoning holds for adding components from the physical layer to the EA. Further research on strategies, characteristics, phases, and measurements of EA resilience may be drawn from a literature study of Aldea et al. [121].

Along with this argument, the context of an organization is simply different from the context in which it operates. Generally, the development of a reference architecture is based on numerous scenarios. However, this implies (1) the existence or availability of these scenarios (e.g., in literature), (2) that system components are known (at least at a generic level), and (3) systematic reuse of common functionality. In turn, this would suggest that applicability is limited to known types of CPSoSs. We call for further research on complex CPSoS enterprise contexts, such as case studies. Inspiration may be drawn from EA research on complex systems [122,123]. Last, the proposed reference architecture in this study is simply positioned as an artifact within a design science cycle. More validation of the reference architecture is needed in follow up research.

### 7.4. Case Study

As with all case studies, the limitation of this research lies in the fact that only one case study was investigated and therefore generalization of the findings has to be taken with caution. Nevertheless, the logistics case study considered was shaped based on previous and ongoing research and discussions with project partners, involving various professional backgrounds. The mapping of the case study by using the reference EA revealed that the architecture played a critical role in steering the project partners towards decision-making and the design of an artifact for the problem at hand. The process also intrigued project partners which were not that familiar with the logistics domain, thereby enhancing the generalizability of our proposed EA.

Although the numerical results and analysis indicated that there is a relation between micro- and macro-goals with respect to some resilience metrics in a CPSoS context, the attempt to break down the case study into numerical categories can undermine the essential richness of the projected capabilities and interrelationsips we were interested in. Therefore, presenting much of the findings of this study in numerical form can be problematic for the validity. For example, we tried to look beneath the umbrella construct resilience, and analyzed the extent to which disruptions are impacting a local and global KPI and how these disruptions might be mitigated. This lack of precision is often neglected by researchers.

With respect to the used definition of resilience in Section 3, we also devoted limited attention to the implementation of how the system exactly is reorganizing, changing, and learning from the disruptive event. Instead, we focused on a functional level of description and considered the occurring phenomena as measured by performance indicators to be an emergent property of a complex system. Nevertheless, we believe that our reference EA is generic with respect to being applicable on many domains which may each bear their own resilience metrics. For example, in the construction industry one may reason differently about resilience than in healthcare processes. Appropriate further empirical research is needed to study the presence of resilience within a more holistic view.

Furthermore, it may seem that the selected performance indicators make little sense separated out from the whole CPSoS concept and proposed reference architecture. With a scope aiming at both SoSs and CPSs, several other related aspects have been given little attention as well. This may render our case study findings as of little value. However, identifying clear boundaries around a CPSoS while at the same time providing exact specifications of a representative case study can be challenging, yet still establishing a provisional truth until contradictory findings or better theorizing has been developed. With providing both a reference EA and an instantiation of the EA, many researchers and practitioners may empathize with our study, because they recognize the sorts of situation that we portray either on a higher level with some simplifications or a more detailed exemplification.

Thus, even though some of the case study findings may be specific to the established logistics domain, it is believed that this study’s validity does not entirely depend upon the case from which we draw the architectural instantiation. To a certain extent, the case study is representative of the trend of CPSoS implementations in complex enterprises. Further research is needed to investigate how enterprises can apply CPSoS functionality affecting the resilience of an enterprise. This will augment current theories and practices of Industry 4.0 and further enrich the proposed reference architecture. A deeper investigation and specification of the CPSoS design principles is also needed.

## 8. Conclusions and Future Work

This paper presented an EA for detection and monitoring of emergent behaviors in a CPSoS context, which enables an integration of distributed autonomous business logic. The purpose of this study was to present an overview of functional building blocks that are required to support the execution and control of business logic for monitoring and detecting emergence. We focused on a means to achieve resilience by uniting the behavior of local autonomous entities with enterprise-wide goals and behaviors. The proposed architecture attempts to target sources of disruptions, such as unexpected emergence, while evolving the CPSoS in a dynamic way. We put forward that facilitating these features is key to obtaining a resilient enterprise “by design”.

Based on a SLR, we identified requirements to be addressed in modeling of the architecture. We presented a reference architecture to guide the process of planning and designing the functionalities within an enterprise. The feasibility of our approach is demonstrated with a proof-of-concept implementation (an instantiation of the EA) through a logistics case study. Experimental results showed, among others the ability to achieve multiple objectives, both micro and macro, by uniting heterogeneous autonomous decision-making units into enterprises, and vice versa; and the ability for enterprises to dynamically adjust business rules of interest, in order to re balance emergent behaviors at the micro- and macro-levels. Results of the latter one indicated the usefulness when proposing disruption mitigation strategies. Understanding emergent behavior as an outcome of an enterprise CPSoS’s complexity is key for achieving a resilient enterprise.

This work has several remaining challenges to be addressed before the full potential of satisfied architectural requirements can be realized in practice. First, although supported by a methodology, the requirement selection required domain knowledge and expertise. We expect that validating our architectures in real-life settings might lead to the identification of other requirements to be added to the set we have at this time. For example, new ways to mitigate unexpected emergence might be of interest. Second, we proposed an instance of an EA based on selected requirements only, while it would be interesting to validate the functionality and proposed artifact more comprehensively. One may incorporate design decisions for this EA instance within a broader decision framework model for SoSs, such as the one illustrated by Raman and D’Souza [69].

The outcome of this work can also spark new research interests. We can explore the possibility of having enterprises self-adapt to contextual changes, thereby more closely embracing the CPS paradigm as well as becoming a time- and self-aware architecture. Another direction would be to integrate the architecture with a SoS dynamic architecture, also including security aspects. Furthermore, we only addressed a case study and instantiation of the proposed EA. The proposed reference architecture may also be applicable to other use cases and industries to evaluate the accuracy of the conclusions discussed above. For example, in further work one may explore different micro–macro integration strategies. A real-life multimodal logistics case study integrated with the smart grid may be an interesting next step. Furthermore, more research is needed on emergent behavior detecting, monitoring, and managing. To this end, analysis of simulations could provide key insights into emergent behaviors of the CPSoS. We also recommend more research on the concept of enterprise resilience and associated terms, such as (anti)fragility, robustness, and flexibility in the context of an enterprise CPSoS. Last, further explorations to leverage united CPSoS, Industry 4.0, and IoT implications by embedding the human’s expertise may be a promising next step. Refined collaborative interactions between humans and CSs inspired from the Industry 5.0 vision, can amplify the human and SoS intelligence to meet the uncertain nature and complexity of the real world.

## Figures and Tables

**Table 1:** Contribution of emergent behavior in a Cyber-Physical Systems of Systems (CPSoS). Adapted from the work in [10].

		Consequence		
		*Beneficial*	*Neutral*	*Detrimental*
**Prediction/**	***Expected***	Normal case	Design fact	Avoided by design rules
**Awareness**	***Unexpected***	Positive surprise	Existence fact	Problematic case
